# Quantitative Evaluation of the Mitochondrial Proteomes of *Drosophila melanogaster* Adapted to Extreme Oxygen Conditions

**DOI:** 10.1371/journal.pone.0074011

**Published:** 2013-09-12

**Authors:** Songyue Yin, Jin Xue, Haidan Sun, Bo Wen, Quanhui Wang, Guy Perkins, Huiwen W. Zhao, Mark H. Ellisman, Yu-hsin Hsiao, Liang Yin, Yingying Xie, Guixue Hou, Jin Zi, Liang Lin, Gabriel G. Haddad, Dan Zhou, Siqi Liu

**Affiliations:** 1 Chinese Academy of Sciences Key Laboratory of Genome Sciences and Information, Beijing Institute of Genomics, Chinese Academy of Sciences, Beijing, China; 2 University of Chinese Academy of Sciences, Beijing, China; 3 Department of Pediatrics, Division of Respiratory Medicine, University of California San Diego, San Diego, California, United States of America; 4 Proteomic Platform, BGI-Shenzhen, Shenzhen, Guangdong, China; 5 National Center for Microscopy and Imaging Research, University of California San Diego, San Diego, California, United States of America; 6 Department of Neurosciences, University of California San Diego, San Diego, California, United States of America; 7 Rady Children’s Hospital, San Diego, California, United States of America; Hertie Institute for Clinical Brain Research and German Center for Neurodegenerative Diseases, Germany

## Abstract

Mitochondria are the primary organelles that consume oxygen and provide energy for cellular activities. To investigate the mitochondrial mechanisms underlying adaptation to extreme oxygen conditions, we generated *Drosophila* strains that could survive in low- or high-oxygen environments (LOF or HOF, respectively), examined their mitochondria at the ultrastructural level via transmission electron microscopy, studied the activity of their respiratory chain complexes, and quantitatively analyzed the protein abundance responses of the mitochondrial proteomes using Isobaric tag for relative and absolute quantitation (iTRAQ). A total of 718 proteins were identified with high confidence, and 55 and 75 mitochondrial proteins displayed significant differences in abundance in LOF and HOF, respectively, compared with the control flies. Importantly, these differentially expressed mitochondrial proteins are primarily involved in respiration, calcium regulation, the oxidative response, and mitochondrial protein translation. A correlation analysis of the changes in the levels of the mRNAs corresponding to differentially regulated mitochondrial proteins revealed two sets of proteins with different modes of regulation (transcriptional vs. post-transcriptional) in both LOF and HOF. We believe that these findings will not only enhance our understanding of the mechanisms underlying adaptation to extreme oxygen conditions in *Drosophila* but also provide a clue in studying human disease induced by altered oxygen tension in tissues and cells.

## Introduction

A constant, stable oxygen supply is essential for cellular respiration, a life-promoting process that is critical for all aerobic organisms on earth. However, many physiological and pathological conditions can induce fluctuations in tissue oxygenation (i.e., hypoxia or hyperoxia). Such fluctuations in oxygen tension may occur in a single context or acute episode (such as in myocardial infarction, ischemic stroke, high-altitude living or oxygen therapy for preterm neonates) or in multiple contexts with sequential episodes (such as in sleep apnea, ischemia-reperfusion or cardiac surgery). As the predominant oxygen-consuming organelles, the mitochondria play an essential role in cellular oxygen homeostasis. For decades, it has been well known that oxygen fluctuation induces dramatic changes in mitochondrial function [1]–[4]. Although mitochondrial adaptation is known to play a critical role in protecting cells from injury and death induced by altered tissue/cell oxygenation [5]–[10], the relevant mechanisms remain largely unknown.

With piled up information and feasible molecular engineering in Drosophila genome, this insect has become a popular model to study physiological functions, including mitochondrial biology. In addition, most cells in *Drosophila* tissues are directly exposed to environmental oxygen because air is directly delivered through a tracheal system that is open to the environment. This unique feature makes *Drosophila* a convenient model in which to study the molecular responses of the mitochondria to oxygen fluctuations. In order to dissect the mechanisms underlying adaptation to hypoxia or hyperoxia, we performed laboratory evolution over the course of several years to generate *Drosophila* strains that tolerated extreme oxygen conditions (i.e., hypoxia-adapted flies (LOF) and hyperoxia-adapted flies (HOF)) [11], [12]. Previous studies on LOF and HOF have shown that metabolic adaptation (through mitochondrial remodeling) plays an essential role in their tolerance to extreme oxygen environments [6], [13]–[15]. For example, we have demonstrated that the decreased activity of respiratory complex II in LOF suppressed oxidative phosphorylation and lowered reactive oxygen species (ROS) leakage, while the attenuation of respiratory complex I and III activity in HOF also lowered ROS generation. These results strongly suggested that the decrease of ROS generation through respiratory adaptation can effectively protect the flies from hypoxia- or hyperoxia-induced injuries regardless of the type of fly (LOF or HOF). Despite intensive efforts to functionally characterize these flies, no direct assessments of the molecular responses to the functional modifications have yet been performed. Although global changes in mRNA levels were observed in the LOF and HOF in our previous studies [11], [12], [14], the corresponding translation products were not examined. Proteins are the effectors of most physiological functions, and mRNA levels are not always correlated closely with protein abundance in living systems [16]–[19], therefore, estimations of the changes in protein abundances in response to extreme oxygen conditions are essential for a better understanding of the molecular mechanisms underlying functional adaptations in mitochondria.

To determine the global mitochondrial proteomic responses to extreme oxygen conditions, we used tandem liquid chromatography coupled with high-resolution mass spectrometry (LC/MS/MS) to survey the mitochondrial proteome. The iTRAQ labeling technique [20]–[24] was used to quantitatively measure relative changes in protein abundances on a large scale. Based on this quantitative proteomic analysis, we measured the global changes in protein abundance in the LOF and HOF mitochondria and provided a solid basis for the further exploration of mitochondrial adaptive mechanisms in extreme oxygen environments.

## Materials and Methods

### Hypoxia- and Hyperoxia-tolerant *Drosophila melanogaster* Strains

The control (C), hypoxia-adapted (LOF) and hyperoxia-adapted (HOF) *Drosophila melanogaster* strains used in current study were generated through long-term laboratory selection as described previously [11], [12]. Flies were cultured in the atmosphere chambers supplied with 4% O_2_ (for LOF) or 90% O_2_ (for HOF) balanced by Nitrogen. Control flies were cultured in the chambers supplied with room air (21% O_2_). All *Drosophila* strains were raised on standard cornmeal food at room temperature. Two biological replicates were analyzed from each group of flies (C1 and C2 for control flies, LOF1 and LOF2 for hypoxia-tolerant flies, and HOF1 and HOF2 for hyperoxia-tolerant flies).

### Stereological Measurement of Mitochondrial Volume Fraction

For mitochondrial volume fraction measurements, total 27 images were analyzed for the Control, LOF and HOF flies as follows. A 9×13 rectangular grid (chosen for ease of use with Photoshop) was overlaid on each image, and mitochondria and cytoplasm lying under intercepts were counted. The relative volume fraction of mitochondria was expressed as the ratio of intercepts coinciding with this organelle relative to the intercepts coinciding with cytoplasm+mitochondria (including the myofibers) and reported as a percentage. Mitochondrial numbers were measured in each image and normalized to the cross-sectional areas of the cells measured with ImageJ (National Institutes of Health).

### Preparation of the Fly Thoracic Muscle Mitochondria

A group of 200∼250 flies were anaesthetized by carbon dioxide, and the thoracic muscle was severed from the head and abdomen on ice blocker. The mitochondria were isolated from the thoracic muscle following the protocol introduced by Ferguson et al [25]. Briefly, the isolated thoracic muscle was pounded gently about ten times to release mitochondria in a tube containing the pre-chilled isolation buffer, 0.32 M sucrose, 10 mM EDTA, 10 mM Tris/HCl, pH 7.3 and 2% (w/v) BSA followed by filtration to remove the tissue particles. After centrifuging at 2200 g at 4°C for 10 min, the pellet was washed twice with the isolation buffer without BSA. Finally the prepared mitochondria were resuspended in the isolation buffer without BSA and stored at −20°C before use.

### Mitochondrial Respiratory Chain Complex Activity Assay

Activities of complex I to IV were measured following previous published protocols. The mitochondrial samples were suspended in 25 mM potassium phosphate buffer/5 mM MgCl2 (pH 7.2) and then subjected to three rounds of freeze/thaw. Complex I: The reaction mix included 2 mM KCN, 2 µg/ml antimycin A, 2.5 mg/ml BSA, 65 µM ubiquinone1 in 25 mM potassium phosphate buffer, and 5 mM MgCl2 (pH 7.2). The activity (NADH: ubiquinone oxidoreductase) was measured after adding 50 µg of mitochondrial sample and 0.13 mM NADH to the reaction mix and following a decrease in absorbance at 340 nm (with reference wavelength at 425 nm). Complex II: The reaction mix included 25 mM potassium phosphate buffer, 5 mM MgCl2, pH 7.2, 20 mM sodium succinate, 50 µm DCPIP, 2 mM KCN, 2 µg/ml antimycin A, and 2 µg/ml rotenone in ethanol. The activity (succinate dehydrogenase) was measured after adding 10 µg of mitochondrial sample and 65 µM ubiquinone1to the reaction mix and following a decrease in absorbance at 600 nm. Complex III: The reaction mix contains 1 mM n-dodecylmaltoside, 1 mM KCN, 1 µg/ml rotenone, and 0.1% BSA in 50 mM potassium phosphate buffer (pH 7.4). The activity (cytochrome c reductase) was measured after adding 10 mM reduced decylubiquinone (DB.H2), 2.5 mM cytochrome c, and 30 µg of mitochondrial protein to the reaction mix and following an increase in absorbance at 550 nm. Complex IV: The reaction mix contains 20 mM potassium phosphate, pH7.0, 0.45 mM n-dodecyl-b-D-maltoside and 15 µM cytochrome c (reduced). The activity (cytochrome c oxidase) was measured after adding 10 mM reduced decylubiquinone (DB.H2), 2.5 mM oxidized cytochrome c, and 4 µg of mitochondrial protein to the reaction mix and following a decrease in absorbance at 550 nm. Each complex activity was presented after normalization to controls.

### Protein Extraction from the Prepared Mitochondria

The prepared mitochondria were dissolved in the lysis buffer containing 7 M urea, 2 M thiourea, 4% (w/v) CHAPS, 20 mM DTT, 1 mM PMSF, 2 mM EDTA and 40 mM Tris-HCl, pH8.8. After sonication with the probe ultrasonicator, the homogenate was centrifuged at 13000 g for 10 min, and an aliquot of the supernatant was taken for determination of protein concentration using Bradford assay.

For the iTRAQ experiments, the mitochondrial proteins were reduced with 10 mM DTT at 56°C for 1 hour, and alkylated with 100 mM IAM at room temperature for 45 min in the dark. The treated proteins were precipitated in acetone at −20°C for 2 hours. After centrifugation at 20000 g for 20 min, the protein pellet was resuspended and ultrasonicated in pre-chilled 50% TEAB buffer with 0.1%SDS. The mitochondrial proteins were regained after centrifugation at 20000 g and the protein concentrations were measured by Bradford assay.

### iTRAQ Labeling to the Mitochondrial Peptides

Total of 150 µg mitochondrial protein in TEAB buffer was incubated with 4.5 µl of trypsin (1 µg/µl) (Promega, Madison, WI, USA) at 37°C for 24 hours in a sealed tube. The completion of tryptic digestion was examined by SDS-PAGE, and the tryptic peptides were lyophilized and dissolved in the 50% TEAB buffer. The protocol of iTRAQ labeling was followed the company manual. The tryptic peptides were incubated with iTRAQ 8 plex reagents (AB Sciex, Foster City, CA, USA) at room temperature for 2 hours, which was dissolved in isopropanol. The labeling efficiency was examined by MS/MS. The mitochondrial peptides from C1, C2, LOF1, LOF2, HOF1 and HOF2 were labeled with iTRAQ reagent 113, 115, 116, 117, 118 and 119 respectively.

### Two-dimensional Chromatography to Separate the iTRAQ Labeled Peptides

At the first dimension, strong cation exchange chromatography (SCX) was used for peptide separation [26]. A Phenomenex Luna SCX column (25 cm x 4.6 mm, 5 µm, 100A) (Phenomenex, USA) was mounted onto the Shimadzu HPLC system with CBM-20A controller, SPD-20A variable wavelength UV detector and LC-6AD pump. Equal amounts of the iTRAQ labeled peptides from all the six samples were mixed, and the peptide mixture was adjusted pH to 3 and was loaded onto the SCX column which was equilibrated with buffer, 10 mM KH2PO4 and 25% acetonitrile, pH 3.0. The peptides were eluted by a 3 steps-gradient program with the elution buffer, 10 mM KH2PO4, 25% acetonitrile and 1 M KCl, pH 3, 0–30% within 20 min, 30–50% in 1 min and 50–100% within 5 min. The flow rate of HPLC was 1 ml/min. The peptide elution was monitored at 214 nm. Total of 30 eluted fractions were collected and concentrated by a lyophilizer for the secondary HPLC.

At the secondary dimension, a nanofluidic h BEH130 C18 (1.7 µm, 100 µmx100 mm) (Waters) column was mounted onto nanoACQuity system (Waters). The collected elution was loaded onto the C18 column followed by desalting with 99% mobile phase A, 98% water, 2% acetonitrile and 0.1% formic acid at a flow rate of 2 µl/min for 15 min. Then the peptides were eluted with a 3 steps elution program with the mobile phase B, 2% water, 98% acetonitrile and 0.1% formic acid at a flow rate of 300 nl/min, 0–5% for 1 min, 5–35% within 40 min and 35–80% within 5 min.

### The Mitochondrial Peptides Identified by TripleTOF MS

A TripleTOF 5600 System (AB SCIEX, Concord, ON), packed with a Nanospray III source (AB SCIEX, Concord, ON) and a pulled quartz tip (New Objectives, Woburn, MA), was used for peptide identification. The ion spray voltage was set at 2.5 kV, the interface heater temperature was set at 150°C, and the curtain gas and nebulizer gas were delivered at 30 PSI and 15 PSI, respectively. For TOF MS scan, resolving power (RP) was greater than or equal to 30000 fwhm. For information dependent acquisition (IDA), survey scans performed in 250 ms, once the detection of ions over a threshold of 120 counts per second and with a 2+ to 5+ charge state, the product ion scans were collected to as many as 30. The time of total cycle was fixed to 3.3 s. Q2 was operated with transmission window of 100 Da for 100%. For collision-induced dissociation of all precursor ions, the sweeping collision energy was set to 35±5 eV and combined with iTRAQ adjust rolling collision energy. The acquired raw data (.wiff) was converted to a data (.mgf) by AB SCIEX MS Data Converter V1.1 software.

### Data Processing

The data files of each fraction were combined together to perform searching against *Drosophila melanogaster* protein database (dmel-all-translation-r5.39) using Mascot software (Matrix Science, Boston, USA). The searching parameters were set as, 1 missed cleavage, carbamidomethylation of cystein as fixed modification, N -terminal iTRAQ8plex mass addition and iTRAQ8plex mass addition on lysine, N-terminal pyroglutamylation, oxidation of methionine and iTRAQ8plex mass addition on tyrosine as variable modifications, peptide tolerance <0.05 and MS/MS tolerance <0.05.

The software of Scaffold (version 3.5.1, Proteome Software Inc., Portland, OR, USA) was employed to calculate iTRAQ quantitation with the Mascot files. In order to achieve high quality MS signal for quantification, the peptides served as the quantitative evaluation should meet three criteria, 1) the peptide thresholds is set ≥80%, the protein threshold is set ≥90%, 2) a target false discovery rate (FDR) threshold is set ≤0.5% at the peptide level, and 3) a protein contains at least two tag-labeld unique peptides.

### Statistical Analysis

The quantitative data derived from the MS/MS intensities of the reporter tags were statistically analyzed by Scaffold with Mann-Whiney test. For each set of the iTRAQ data, the MS/MS intensities were globally normalized by the MS signals of control flies. For multiple comparisons among the fly groups, an identified protein gained 4 tag intensity ratios with the corresponding p values when two parallel pairs of samples were compared, such as LOF1/C1, LOF1/C2, LOF2/C1 and LOF2/C2 for hypoxia tolerant group, and HOF1/C1, HOF1/C2, HOF2/C1 and HOF2/C2 for hyperoxia tolerant group. A protein defined as differential protein should match with 2 criteria: 1) one of the 4 ratios was >1.5 or <0.67 with p<0.05; and 2) among 4 ratios, at least 3 ratios were at the same trend with significant difference (p<0.05).

### Multiple Reaction Monitoring (MRM) Confirmation of Some Proteins from Complex I

All of the MRM confirmation experiments were conducted using the same experimental condition reported by Zhang [27] with some modifications. Approximately, 3 µg of thoracic muscle proteins were digested by trypsin, and the digested peptides were loaded onto a nanofluidic h BEH130 C18 (1.7 µm, 100 umx100 mm) column (Waters). The separated peptides were identified by TripleTOF 5600 System (AB SCIEX, Concord, ON, USA) at IDA mode, and the MS/MS data was treated with ProteinPilot (AB SCIEX, Framingham, MA, USA) and Skyline 1.4 (MacCoss Lab, University of Washington, WA, USA). On the basis of the iTRAQ and label free proteomics, several target proteins which responded to oxygen stress were selected. The ideal peptides for MRM from the target list were further picked up upon the criteria, 1) unique peptide for a protein, 2) no cysteine or methionine, 3) 7–25 amino acids, and 4) no miss cleavage of trypsin. All MRM analysis was performed on a QTRAP5500 mass spectrometer (AB SCIEX, Framingham, MA, USA.) equipped with a nanoAquity system (Waters, Milford, MA) with the parameter settings as ionspray voltage (IS), 2500 V, curtain gas (CUR), 35.00, ion source gas1 (GS1), 20.00, ion source gas 2 (GS2), 0.00, collision gas (CAD), high, interface heater temperature (IHT), 150, entrance potential (EP), 10.00, Q1 and Q3, unit resolution. All the samples, C, LOF and HOF, were consisted with that mentioned with the iTRAQ analysis.

The raw data of MRM was processed by Skyline 1.4. After manual inspection and retention alignment, the peaks filtered with S/N>10 were qualified for quantification. The sum of all the transition areas of a protein was used to indicate the protein abundance.

## Results

### Phenotypic Changes in LOF and HOF Mitochondria

*Drosophila melanogaster* strains that tolerate extremely low or extremely high oxygen conditions have been generated by long-term laboratory evolution over many generations. LOF flies can live in normally lethal hypoxic conditions containing as little as 4% O_2_, and HOF flies can live in normally lethal hyperoxic conditions containing as high as 90% O_2_ [11], [12].

**Figure 1:**
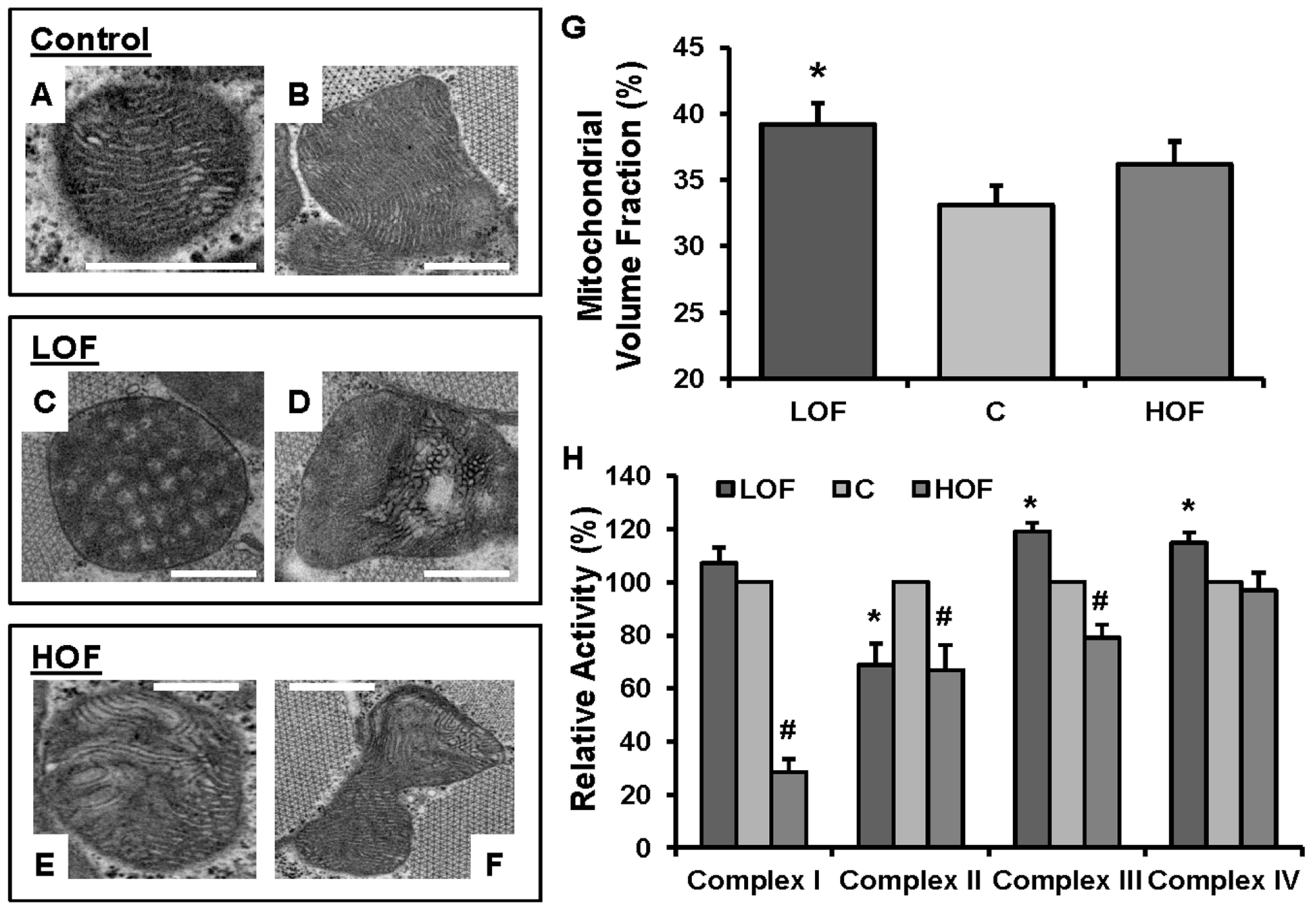
Morphological and functional characterization of control, LOF, and HOF thoracic muscle mitochondria. (A and B) Representative electron micrographs of thoracic muscle mitochondria from control flies. (scale = 500 nm) (C) Honeycomb-patterned mitochondria from LOF (volume fraction = 23.1±5.7%) (scale = 500 nm). (D) Mitochondria with large-amplitude swelling (the transparent “empty” spaces) from LOF (volume fraction 6.8±1.8%) (scale = 500 nm). (E and F) Mitochondria with microswelling inside the cristae and regional distortion; some of the distorted cristae were rounded (circular profile), from HOF flies (volume fraction 8.0±1.2%) (scale = 500 nm). (G) Mitochondrial volume fraction of control, LOF, and HOF. A significant increase in mitochondrial volume fraction was detected in the LOF samples (* p<0.01, n = 9). Each bar represents the mean ±SEM of 9 repeated measurements. (H) Activities of respiratory chain complexes in mitochondria isolated from control, LOF, and HOF thoracic muscle. Compared to the control, the activity of LOF respiratory chain complex II was reduced; however, the activities of complexes III and IV were increased (* p<0.05, n = 6). In HOF, the activities of complexes I, II and III were reduced, and the activities of complex IV was unchanged (# p<0.05, n = 6). All of the activities are presented as percentages of the control values (mean ±SEM).

We used transmission electron microscopy (TEM) to observe distinct morphological changes in the mitochondria of LOF and HOF flies. Representative electron micrographs of thoracic muscle mitochondria from the control, LOF and HOF samples are shown in Figure 1A to 1F. These micrographs revealed that the mitochondrial structures in LOF and HOF changed significantly comparing with control; differences were primarily observed in the cristae, but different types and extents of morphological changes were observed. The LOF mitochondria were representative of the two major abnormal classes: the first class is the honeycomb-patterned mitochondria (volume fraction 23.1±5.7%, Figure 1C) as reported before [28], and the second class is with large amplitude swelling (as indicated by the transparent “empty” spaces) (volume fraction 6.8±1.8%, Figure 1D), which can be found in myocardial ischemia in mammals [29], [30]. However, these two structural aberrations were not observed in the HOF. Instead there was microswelling inside the cristae and regional distortion; sometimes the distorted cristae were rounded (circular profile) (volume fraction 8.0±1.2%; Figure 1E and 1F). Moreover, a significant increase in the mitochondrial volume fraction compared to controls was detected in the LOF (p<0.01) but not in the HOF (Figure 1G). Generally, cristae contain many respiratory chain complexes and membrane-associated proteins, and changes in cristae branching and surface area alters mitochondrial respiration efficiency. Therefore, mitochondrial structural distortions may reflect changes in mitochondrial proteins during adaptation to extreme oxygen environments.

Previously, to understand whether LOF or HOF display adaptive responses in mitochondrial biochemistry, we measured the activities of the four respiratory chain complexes in LOF and complexes I and III in HOF [6], [15]. The results of this analysis are presented in Figure 1H: 1) the activity of complex I in the LOF was comparable to that of the control group, whereas that in the HOF was dramatically decreased, 2) the activity of complex II was decreased in both the LOF and HOF, and 3) the activities of complex III and IV were increased in the LOF but a decrease in complex III activity was observed in the HOF. Electron flow during mitochondrial respiration is commonly driven by two basic pathways. NADH (complex I) and FADH2 (complex II) converge on the same respiratory reaction through complexes III and IV, while the respiration rates are dominated by the NADH pathway [31]. The respiratory activities shown in Figure 1H indicate that, under extreme oxygen conditions, the respiratory regulation through the FADH2 pathway was similar in the mitochondria of both strains, whereas the regulation through the NADH pathway was very different between the LOF and HOF. Specifically, the enzymes involved in complexes I and II in the HOF and in complexes III and IV in the LOF may play key roles in adaptive regulations.

### Quantitative Proteomic Profiling Experimental Design

A fine regulation of mitochondrial functions is well known to be detrimental to metabolic adaptations to environmental oxygen fluctuations. Because mitochondria are heavily enriched in skeletal muscle cells, and the function of muscles is tightly regulated by the oxygen supply, we examined fly thoracic muscle mitochondria isolated from LOF, HOF or control samples to perform global and quantitative analyses of the proteomic responses to extreme oxygen conditions in mitochondria.

**Figure 2:**
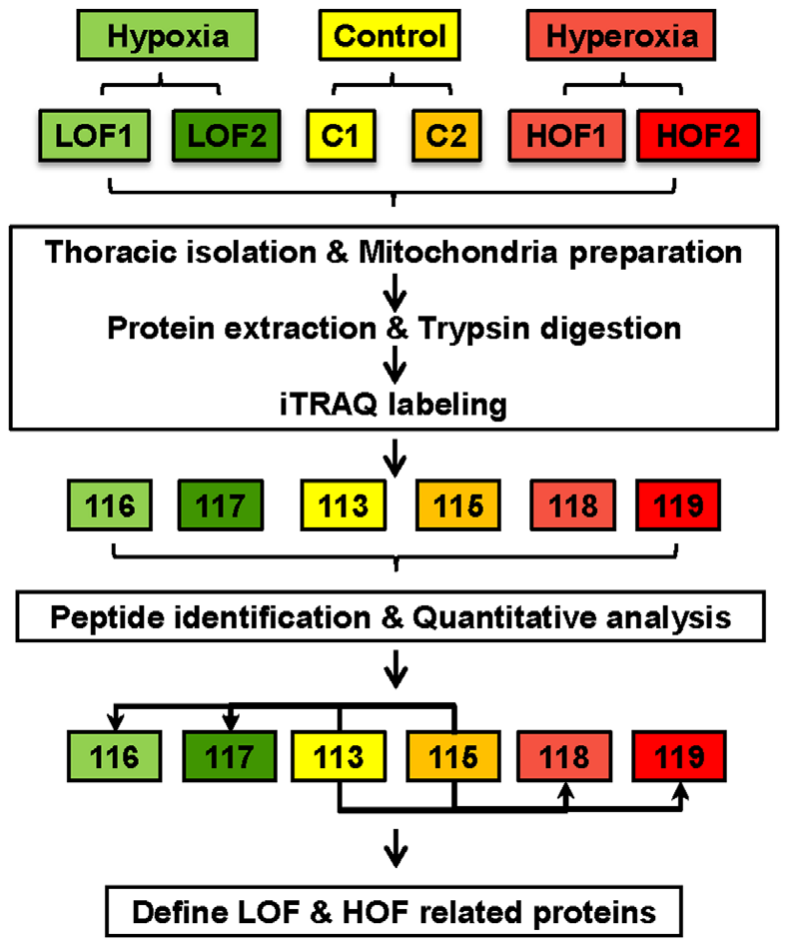
Schematic illustration of the experimental workflow and overview of the proteomic experiments. To characterize the mitochondrial proteomes of control (C), hypoxia-adapted flies (LOF), and hyperoxia-adapted flies (HOF), a MS-based experiment was performed. Thoracic muscle mitochondrial samples were isolated from control, LOF, and HOF. Proteins were extracted and digested with trypsin. Peptides were labeled using iTRAQ tags, equally mixed, and then pre-fractionated using strong cation exchange (SCX). Two biological replicates were performed for each experiment. SCX fractions were analyzed by high-resolution LC-MS/MS. Differentially expressed proteins were identified by comparing LOF and C (for differentially expressed proteins in LOF) or HOF and C (for differentially expressed proteins in HOF).

This proteomic survey was broadly divided into three phases, as illustrated in Figure 2. First, the mitochondrial samples were isolated from the thoracic muscles of the LOF, HOF, and control groups. Duplicated samples were isolated from each group and labeled as C1 and C2 (control group), LOF1 and LOF2 (LOF group) and HOF1 and HOF2 (HOF group). Each sample contained mitochondria pooled from approximately 150 flies. The second phase involved the iTRAQ labeling of trypsin-digested peptides. The samples were labeled as follows: C1 with 113, C2 with 115, LOF1 with 116, LOF2 with 117, HOF1 with 118, and HOF2 with 119. Third, after mixing equal amounts of the labeled peptides from the 6 groups, the mixed peptides were analyzed by LC/MS/MS. The tag intensity differences between HOF or LOF and the control were statistically evaluated by Scaffold (version 3.5.1, Proteome Software Inc.) (e.g., 116 versus 113 and 114, 118 versus 113 and 114, etc.). Finally, the differentially expressed proteins were called based on the peptide search and quantitative evaluations.

### Quality Evaluations of the MS/MS Data for the Qualitative and Quantitative Analysis of the Fly Mitochondrial Proteins

**Figure 3:**
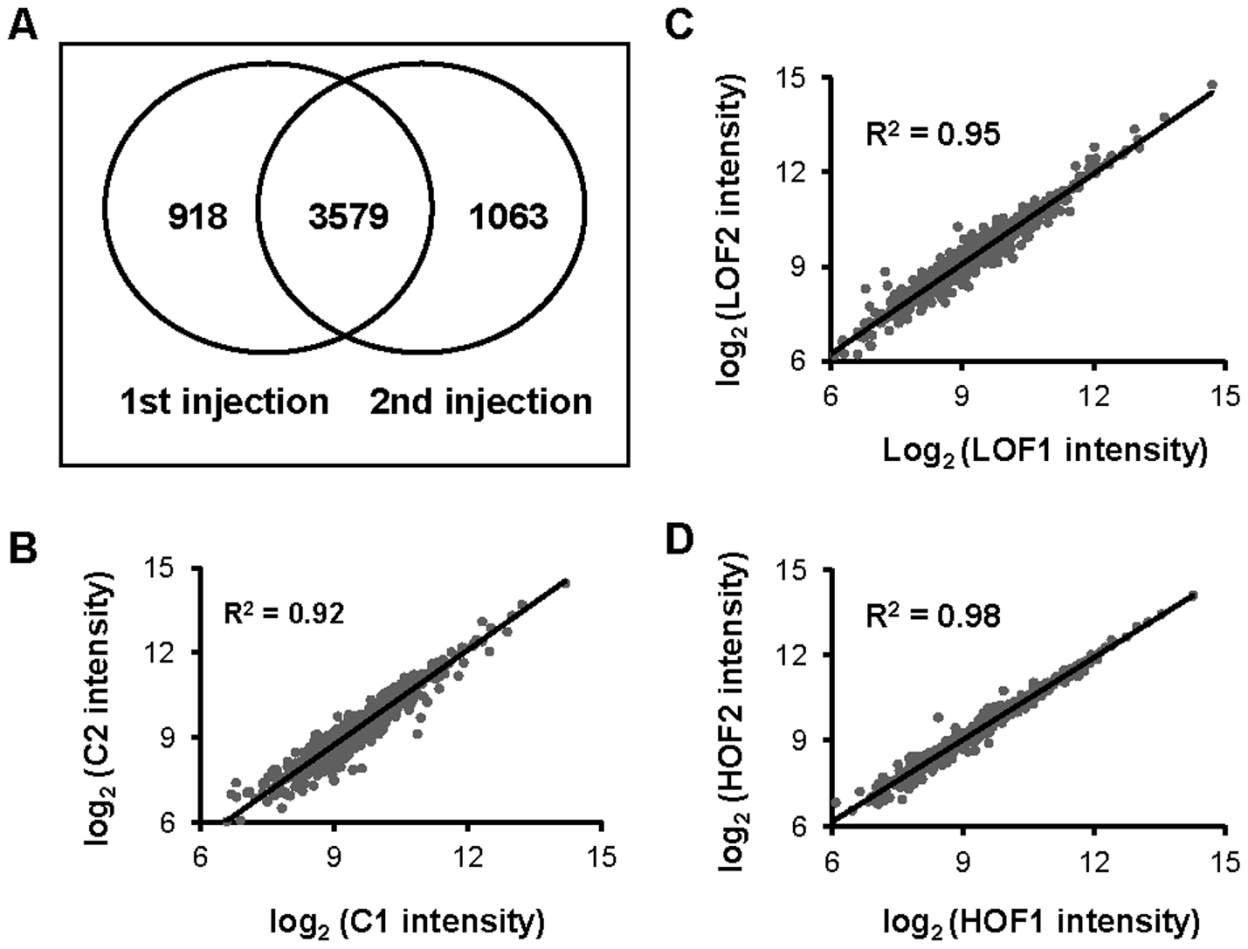
Quality control for the qualitative and quantitative data generated by mass spectrometry. (A) The overlap rate of the unique peptides identified in replicate injections. (B to D) Correlation analysis of the quantitative signals in the biological replicates was performed using the Pearson method. The log_2_ values of the normalized tag intensities of the proteins in one sample are plotted against those of the parallel sample. The squares of the correlation coefficients (R^2^) and the slope of the regression curve are listed in the insert. B: Control; C: LOF; and D: HOF.

The digested peptide pool, which contained equal amounts of iTRAQ-labeled peptides collected from the LOF, HOF and control groups, was loaded onto the SCX HPLC instrument and separated into 36 elution fractions. Based on the peptide distributions in these fractions, the eluted fractions were further pooled into 15 fractions and then reloaded onto a nano RP HPLC column coupled with a TripleTOF 5600 MS system with dual injections. The qualitative and quantitative proteomics data are summarized in Figure 3 and Table S1. Based on the principle that a protein can be identified by at least two unique peptides, a total of 718 proteins from 5426 unique peptides corresponding to 40020 MS/MS spectra (FDR<0.01) were identified. The overlap rate of the identified proteins in the parallel injections was approximately 85% (data not shown). Importantly, the reproducibility of the peptide identification in the parallel injections was approximately 78% (Figure 3A and Table S2–S4). The high reproducibility of the identified peptides/proteins reflects the high quality of the MS data, assuring the accuracy of the qualitative protein calling. In addition, more than 99% of the identified peptides were labeled with the tags (data not shown), indicating the completion of the labeling reactions and that the tags were representative of the identified peptides.

To confirm that the quantitative data from the labeled tags were reproducible, the Pearson correlation was used to compare the protein quantification results between the duplicated mitochondrial preparations. The slopes of the linear regression were 1.12, 0.95, and 0.96, and the correlation coefficients of the duplications were 0.92, 0.95, and 0.98 for the control, LOF, and HOF samples, respectively (Figure 3B to 3D). Between the parallel duplicated samples, more than 99% of the fold changes in the normalized protein intensities were smaller than 1.2-fold; less than 1% of the fold changes were between 1.2- and 1.5-fold, and no change was larger than 1.5-fold. These data strongly demonstrated the high reproducibility of the tag intensities that were qualified for further quantitative analysis.

We established four steps for identifying proteins with significant differences in expression among the groups. First, proteins were required to have at least two unique peptides labeled with iTRAQ to qualify for quantitative analysis. Second, the correction of isotope impurities and the normalization of the intensity median were performed. Thirdly, among all four alternative comparisons of LOF or HOF versus the control, a differentially expressed protein should appear with similar abundance levels at least three times. Finally, because the mitochondrial preparation was not purified, any obvious contaminating proteins from other organelles were excluded from the list of differential proteins. According to the results of the Mann-Whitney test, the threshold of significance for fold changes in relative abundance between the LOF or HOF samples and the control was ≥1.5 fold, with a p-value <0.05 (Table S5–S7). Following all the analyses described above, 115 unique proteins were identified as being differentially expressed (55 in LOF and 75 in HOF). Among these proteins, 15 exhibited differential expression in both the LOF and HOF samples.

### Correlation Analysis of the Differential Proteins and the Corresponding mRNAs in LOF and HOF

**Figure 4:**
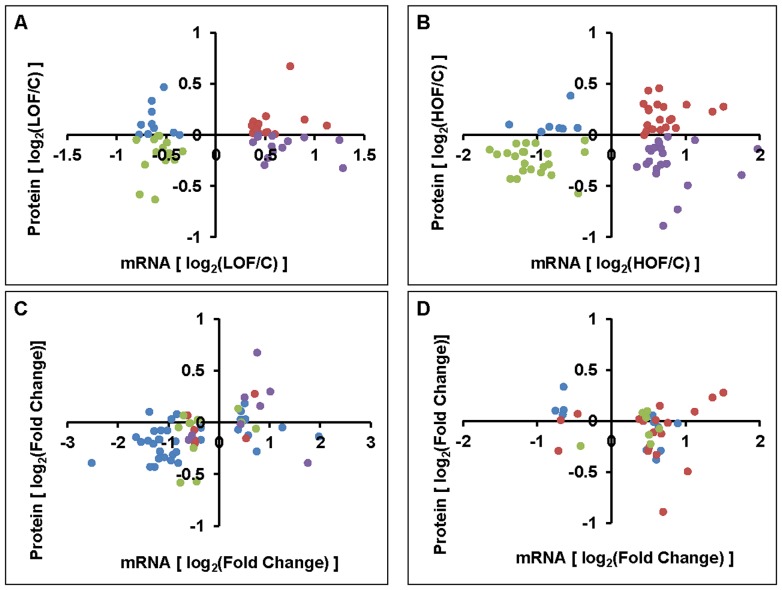
Correlation analysis towards the differential proteins and corresponding mRNAs in LOF and HOF. (A and B) Scatter plot correlation analysis of the differentially expressed proteins and corresponding mRNAs in LOF (A) and HOF (B). The log_2_ values of the normalized fold changes for the differential proteins are plotted against those of the corresponding mRNAs in LOF or HOF. The spots with different colors represent the four modes: red, up-regulated protein and mRNA; green, down-regulated protein and mRNA; blue, down-regulated protein but up-regulated mRNA; and purple, up-regulated protein but down-regulated mRNA. (C and D) Scatter plot correlation analysis of the differentially expressed proteins and corresponding mRNAs in certain functional groups (C, proteins uniquely localized to the mitochondria; D, mitochondria-associated proteins). Different colors represent different functional groups. 4C: blue, respiratory chain proteins; red, mitochondrial translation; green, carbohydrate/lipid metabolism; and purple, chaperones. 4D: blue, calcium regulation; red, membrane-associated; and green, amino acid synthesis/metabolism.

It is known that protein abundance does not always correlate with the corresponding mRNA level, particularly for mRNAs and proteins of medium and low abundance [32]. According to our previous reports, the mRNA levels of LOF and HOF adapted to hypoxia or hyperoxia [11], [12]. Herein, we asked whether these oxygen-sensitive mitochondrial proteins and their corresponding mRNAs followed a similar pattern in the two fly models. To evaluate these correlations, we compared the quantitative mRNA expression data from LOF or HOF obtained from expression arrays in our previous studies [12], [14] with the protein data obtained in this study. Among the 115 differentially expressed mitochondrial proteins, 110 (96%) were covered by the expression array, including 53 proteins from LOF and 71 from HOF. Using a scatter plot, the Log_2_ abundance of the differentially expressed proteins was plotted against the Log_2_ abundance of the corresponding mRNAs. In this plot, genes with similar changes in protein and mRNA abundance were located in the 1^st^ or 3^rd^ quadrants of the plot, whereas genes with opposing trends in mRNA and protein abundance changes were located in the 2^nd^ or 4^th^ quadrants, as illustrated in Figure 4A and 4B. Among the 53 proteins that were differentially expressed in the LOF, 29 (55%) exhibited the same pattern as their corresponding mRNAs, including 14 up-regulated (1^st^ quadrant) and 15 down-regulated (3^rd^ quadrant) proteins (Figure 4A, Table S8). Among the 71 proteins that were differentially expressed in the HOF, 43 (61%) exhibited the same pattern as their corresponding mRNAs, including 20 up-regulated (1^st^ quadrant) and 23 down-regulated (3^rd^ quadrant) proteins (Figure 4B, Table S8). However, 45% of the differentially expressed proteins in the LOF and 39% of those in the HOF did not exhibit the same patterns as their corresponding mRNAs, implying that the changes in the abundances of these proteins were not regulated at the transcriptional level.

Furthermore, we investigated whether the mitochondrial proteins, such as respiration- and tricarboxylic acid (TCA) cycle-related proteins, that were differentially expressed in response to extreme oxygen stress exhibited the same pattern of expression as their corresponding mRNAs. As shown in Figure 4C and Table S9, there was a strong correlation between the protein and mRNA abundances of this set of mitochondrial factors; this group included 27 of the 35 respiration-related proteins, 4 of the 6 proteins related to mitochondrial translation, 8 of the 12 proteins related to carbohydrate/lipid metabolism, and 6 of the 8 proteins related to chaperone functions. Overall, 74% of these proteins exhibited the same trend of changes in both protein and mRNA abundance. However, as shown in Figure 4D and Table S9, the abundances of some proteins and their mRNAs were poorly correlated; these proteins included 1 of the 11 proteins related to calcium regulation, 7 of the 18 membrane-associated proteins, and 4 of the 7 proteins involved in peptide/amino acid metabolism.

### Analysis of Functional Categorization of the Differential Proteins in LOF and HOF

Based on an analysis of the Cluster of Orthologous Groups (COG) ‘organelle’ annotations (flybase.org), all of the fly proteins identified were broadly sorted into the following 8 groups: 34.3% mitochondria, 22.5% membrane, 7.5% lipid particle, 6.2% endoplasmic reticulum, 5.0% cytoplasm, 4.2% nucleus, 1.1% extracellular and 19.3% unknown. Based upon the information obtained from databases and from the literature, we excluded 123 typically non-mitochondrial proteins from the list of identified proteins, leaving 588 fly mitochondrial proteins for further analysis. The 588 mitochondrial proteins identified in the current study included all 231 mitochondrial proteins that were previously reported by Alonso et al., who used two-dimensional electrophoresis coupled with mass spectrometry [33]. The proteins involved in respiration complexes, TCA cycle, and ATP synthase complexes are typical mitochondrial proteins. The data in Table S10 show that 62% of respiration complex components, 59% of TCA enzyme components, and 52% of ATP synthase complex components were identified in the proteomic analysis.

**Figure 5:**
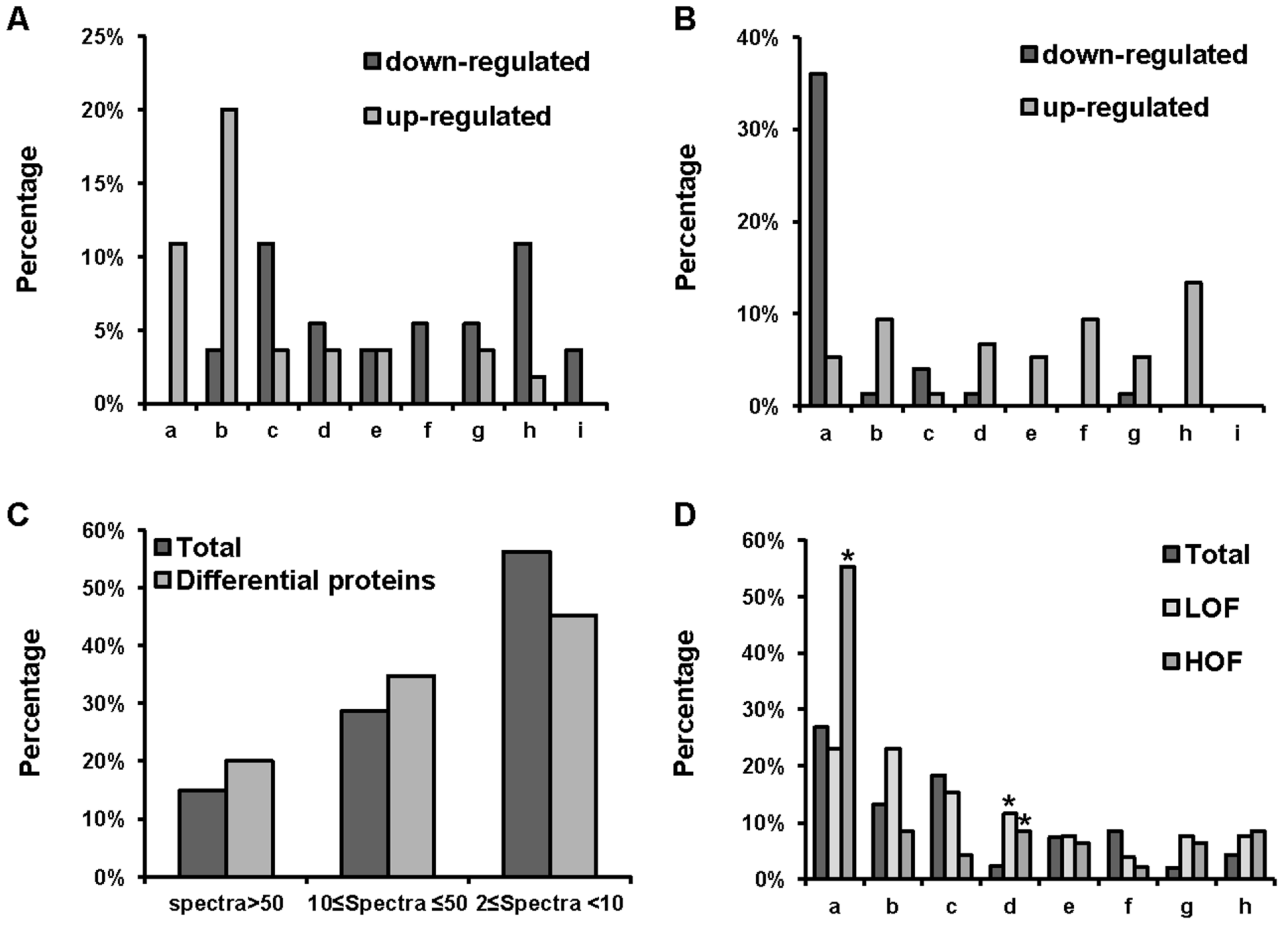
Functional category and protein abundance distribution analyses. (A and B) The distribution of proteins with differential expression in LOF or HOF across functional categories. The proteins were categorized in 9 groups as follows: a, Respiratory chain complex proteins; b, Membrane-associated proteins and transporters; c, Carbohydrate/lipid metabolism; d, calcium regulation-related; e, Peptide or amino acid synthesis/metabolism; f, Oxidation-sensitive; g, Chaperones; h, Translation-related; and i, Detoxification-related. Black bars: down-regulated proteins; gray bars: up-regulated proteins. (C) The abundance distributions of all mitochondrial proteins identified and the differentially expressed proteins. The protein abundance was categorized according to the total MS/MS spectra per protein and generally divided into three abundance groups (high: >50 spectra; medium: 10≤50 spectra; low: <10 spectra). The black bars represent the total mitochondrial proteins identified, and the gray bars indicate the proteins with differential expression in LOF or HOF. (D) The distribution of the abundant proteins across different protein functional categories. The abundant proteins were characterized as proteins with >10 total MS/MS spectra; the total mitochondrial proteins identified and the differentially expressed proteins in LOF and HOF were analyzed. The proteins were categorized in 9 groups as described above. The black, light-gray and dark-gray bars represent the total mitochondrial proteins, the differentially expressed proteins in LOF and HOF, respectively. The percentage of respiratory chain complex proteins in HOF was significantly larger (* p<0.05) than that of the control and LOF. The percentages of calcium-regulation-related proteins in LOF and HOF were both significantly larger (* p<0.05) than that of the control.

We next inquired whether the differentially expressed mitochondrial proteins were associated with physiological and biochemical functions that could be associated with the LOF and HOF’s tolerance of such harsh oxygen conditions. Following the COG analysis, the differentially expressed proteins were divided into 9 functional categories: calcium regulation-related, respiration-related, chaperone-related, carbohydrate metabolism-related, peptide and amino acid metabolism-related, membrane-associated, detoxification-related, oxidation-sensitive, and translation-related proteins. As shown in Figure 5A and 5B, most proteins in the calcium regulation-related, oxidation-sensitive, chaperone-related and translation-related groups were less abundant in LOF. In contrast, the differentially expressed proteins were generally more abundant in HOF, with the exception of the respiration complex proteins, which exhibited decreased abundances. Interestingly, a comparison of the functional categories between LOF and HOF revealed that the proteins in five groups (respiration, calcium regulation-related, oxidation-sensitive, chaperon and translation-related) displayed opposite responses. For example, the majority of the respiration complex proteins had higher abundances in LOF than in the controls, whereas most of these proteins showed reduced abundance in the HOF samples. These results suggest that the respiratory capacity in HOF may be lower than that in LOF, implying that distinct mitochondrial mechanisms were modulated to survive under extremely hypoxic or hyperoxic conditions.

To determine whether the changes in protein levels were correlated with absolute protein abundance, we divided the differentially expressed proteins into three groups based on the sum of the MS/MS spectrum counts per protein as follows: proteins with spectrum counts larger than 50 were defined as high-abundance proteins, those with spectrum counts from 10–50 were defined as medium-abundance proteins, and those with spectrum counts less than 10 were defined as low-abundance proteins. We compared the distributions of the three abundance groups in the complete group of identified mitochondrial proteins and the differentially expressed proteins in LOF and HOF. As shown in Figure 5C, the proportions of the identified mitochondrial proteins in the high-, medium-, and low-abundance groups were 15%, 29% and 56%, whereas the proportions of all 115 differentially expressed proteins in the high-, medium-, and low-abundance groups were 20%, 35%, and 45%, respectively. The differentially expressed proteins were enriched with proteins from the high- and medium-abundance groups relative to the group of total proteins, indicating that the proteins with relatively higher abundance were more sensitive to extreme oxygen stress. Considering that protein length may affect the spectrum count, we divided the proteins into several groups based on their lengths; we determined that the proteins in all three groups were evenly distributed among the length groups, demonstrating that the spectrum counts reflected absolute abundance rather than protein size (Figure S1). We additionally categorized all of the mitochondrial proteins with sum spectra larger than 10 (either in total or among the differential proteins) according to their biological functions. As shown in Figure 5D, the abundant proteins were categorized into 9 functional groups. The percentages of the proteins in each category against the total proteins of corresponding groups (total, LOF and HOF) were achieved (number of proteins in each group divided by total number of the proteins in corresponding group). Interestingly, the percentage of respiratory chain complex proteins was significantly higher (p<0.05) in HOF than in the control and LOF samples, while the percentage of membrane-associated proteins was higher in LOF than in the other two groups. Furthermore, the abundant proteins were highly represented in the functional categories of calcium regulation, chaperones, and protein translation in both the LOF and HOF samples. Together, these data demonstrated that the more abundant mitochondrial proteins were sensitive to extreme oxygen stresses.

Technically, a higher spectrum count is correlated with a higher quality of statistical estimation in quantitative proteomics. Most of the TCA cycle enzymes identified were ranked at higher abundances (13 out of 15) and were found to be insensitive to low or high oxygen conditions. Indeed, in LOF, only one TCA cycle enzyme with two isoforms (isocitrate dehydrogenases/CG6439 and CG12233) was detected, with 149 and 213 spectra, and exhibited an increased abundance; however, in HOF, succinate dehydrogenase, with 112 spectra, showed a decreased abundance. It is reasonable to speculate that because the majority of the TCA cycle enzymes maintained consistent abundances in the LOF or HOF samples, a single TCA cycle enzyme with a greater change in abundance may directly affect the pathway’s activity. Therefore, our data suggested that the activity of the TCA pathway in LOF was relatively higher than that in HOF. Another important observation was that nearly 40% of the identified proteins in the respiration chain complexes with high spectrum counts (primarily from complex I) displayed reduced abundances in HOF compared to the controls. In contrast, in the LOF mitochondria, although most components of the respiratory chain complexes were relatively stable, several respiration-related proteins displayed altered abundances (primarily from complexes III and IV) in an increased abundance mode. These observations led us to hypothesize that such changes in respiration-related proteins contributed to the inhibition of ETC I and II activities in the HOF samples and the enhanced ETC III and IV activities in the LOF samples (Figure 1H).

### Confirmation of the Differential Proteins by MRM

**Figure 6:**
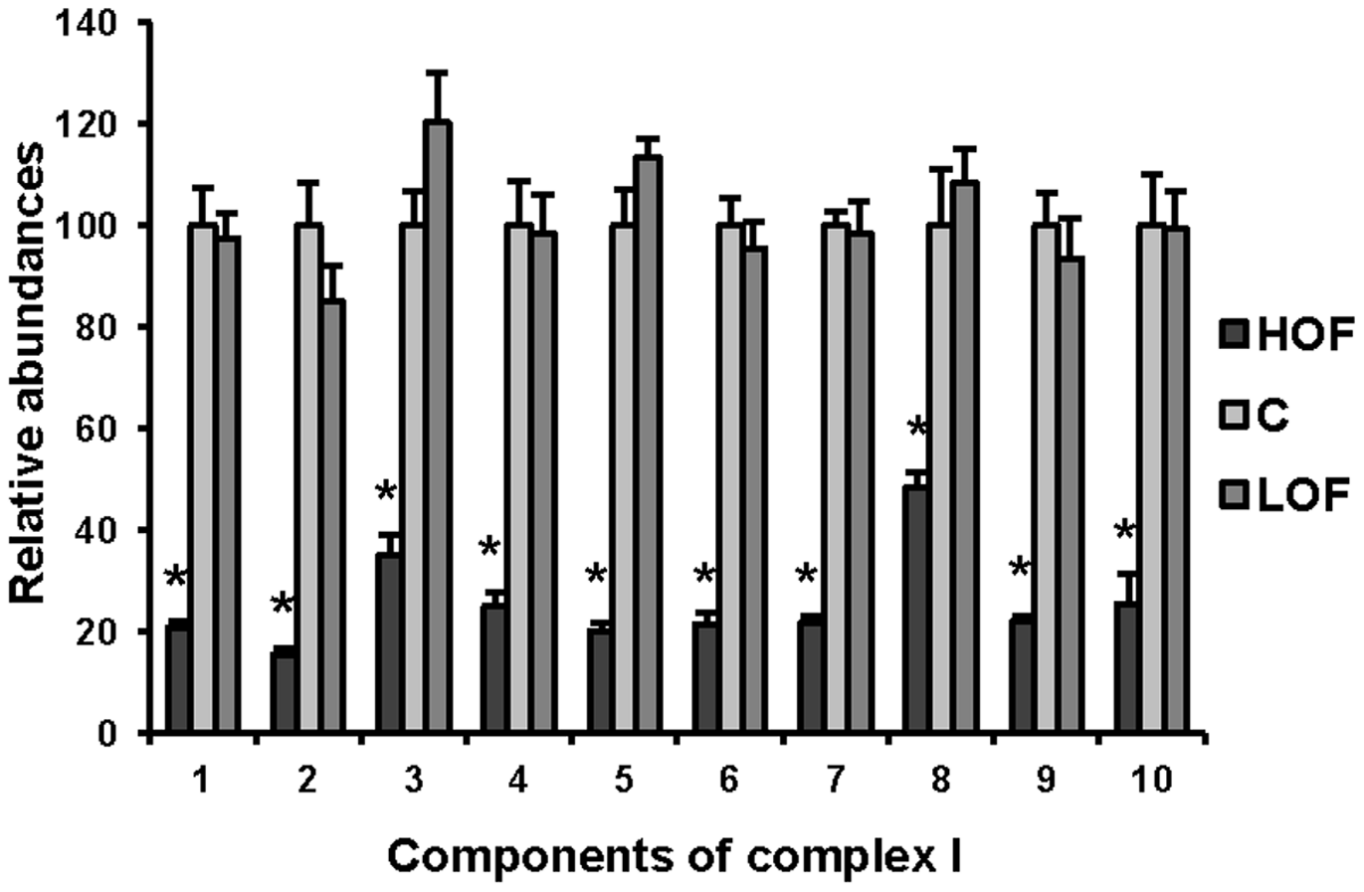
The differential mitochondrial proteins derived from iTRAQ were confirmed by the MRM analysis. The target proteins were selected base on the principles described in the text. The numbers 1–10 in the X axis represent targets as FBpp0071128, FBpp0074481, FBpp0088174, FBpp0078847, FBpp0077425, FBpp0077859, FBpp0070370, FBpp0072231, FBpp0087333 and FBpp0073949 (all from mitochondrial respiratory chain complex I), respectively. The Y axis indicates the abundance ratios of the individual targets that are normalized by the corresponding abundance of control. All the samples came from two biological replicates, and each sample was analyzed by MRM with triplicate. The calculation of abundance averages with standard deviations and the t-test were conducted with Excel (Microsoft Office 2010, USA). Black: HOF; Light gray: Control; Dark gray: LOF. All the abundances of components in HOF were significantly lower (* p<0.05, n = 6) than that of control and LOF.

Even though we adopted the stringent analysis to the quantitative proteomics generated from iTRAQ, the proteomic conclusions were still not convincible enough if lack of additional confirmation by alternative approach. How did we design a set of experiment to confirm the quantitative data derived from iTRAQ? First of all, we should select the proper targets to be confirmed, in which the selected differential proteins existed within the certain functional path as unique mitochondrial proteins and exhibited the distinct abundance between LOF and HOF. Upon such consideration, we selected ten components of complex I as the targets, whose abundances were significantly down-regulated in HOF, but were almost comparable in LOF measured by iTRAQ. Secondly, we should take an appropriate approach to globally quantify the targets. As the commercialized antibodies against the Drosophila proteins were limited and the global quantification for ten proteins were not easily conducted by either Western Blot or ELISA, we finally employed MRM to quantitatively monitor the mitochondrial targets. The protein identification based on the IDA scan in TripleTOF 5600 demonstrated that all the peptides in the ten selected targets were detected with significantly high MS/MS intensities, implying these peptides likely qualified for MRM assay. The comparisons of abundance of ten targets among the fly models shown in Figure 6 clearly revealed that all the targets’ abundance in HOF was significantly lower than that of control and LOF (p<0.05), whereas in LOF was quite comparable with that of control. Carefully analyzing the quantitative data, we found there was a slight difference in the changes of quantitative fold between iTRAQ and MRM. Averagely, 59% abundance decrease in HOF for the ten targets was measured by iTRAQ, while 75% abundance decrease was detected by MRM. Therefore, the conclusions as regards the differential mitochondrial proteins in response to LOF and HOF derived from iTRAQ were well confirmed by MRM.

## Discussion and Conclusion

Mitochondria are involved in a wide array of cellular processes in eukaryotic cells, including energy metabolism, apoptosis, and aging. The mitochondrial proteome is dynamically programmed by nuclear and mitochondrial DNA to adapt its functional capacity to the needs of the tissues and cells exposed to various physiological and pathological conditions. For example, previous studies have shown that mitochondria are reprogrammed in many different clinical conditions, including diabetes [34], obesity [35], cancer [36] and numerous genetic disorders [37]. However, the method by which the mitochondrial proteome adapts to fluctuations in tissue oxygenation is largely unknown. Because hypoxia and hyperoxia are common factors that contribute to clinical conditions in the cardiac system, an understanding of the mitochondrial mechanisms that underlie hypoxia or hyperoxia tolerance will provide valuable information that will aid in the development of novel therapeutic strategies and diagnostic biomarkers. The current study represents the first comprehensive examination and comparison of the mitochondrial proteomes of *Drosophila* that are adapted to life in extreme oxygen conditions.

Insect thoracic muscle has been reported to be a highly metabolically active, containing a large number of mitochondria [38]. To identify primary mitochondrial events related to adaptation to long-lasting extreme hypoxic or hyperoxic stresses, we have examined the pathological consequences of hypoxia and hyperoxia in adult LOF and HOF flies using electron microscopy. We discovered striking patterns reflecting structural alterations in the mitochondria of the LOF and HOF thoracic muscles; these alterations were not detected in the controls. The honeycomb-patterned mitochondria of the LOF samples were originally described in our previous study. The increased cristae branching and surface area detected in the LOF mitochondria indicated that this type of structural rearrangement was an important mechanism of regulation of respiratory activity in extremely oxygen-poor environments [28]. In addition, 2 mitochondrial structural disturbances that were similar to, albeit much less severe than, the changes observed under pathological conditions were observed in LOF and HOF. For example, large-amplitude swelling, which can occur due to myocardial ischemia in mammals [29], [30], was observed in LOF, and mitochondria with regionally swollen and rounded cristae, which were similar to those found in flies that were treated acutely with extremely high oxygen levels, were observed in HOF [39]. Furthermore, changes in the enzymatic activities of the respiratory chain complexes were detected in the LOF and HOF samples. With the exception of the common down-regulation of respiratory complex II, the activity of complex I was down-regulated only in HOF, and the activities of complexes III and IV were up-regulated only in LOF, demonstrating distinct modulations of respiratory capacity in the flies that were adapted to low- or high-oxygen environments. The structural and/or activity changes observed in LOF and HOF strongly suggested that changes in the mitochondrial proteome played an important role in regulating the ability of mitochondria to adapt to environmental oxygen fluctuations.

The mitochondrial proteome is highly dynamic and diverse, including proteins with a broad range of pI, molecular weight, and solubility values. To date, the comprehensive, in-depth identification and quantification of mitochondrial proteins has been a significant challenge. For example, although there are an estimated 2000 to 2500 mitochondrial proteins in the human genome [40], only approximately 600 have been identified at the protein level [41]. Even less is known about the *Drosophila* mitochondrial proteome. To address the challenges related to the enormous dynamic range of proteins in the mitochondrial proteome, we combined iTRAQ labeling with SCX based fractionation and a shotgun proteomic method to quantify the changes in protein abundance in flies adapted to extremely low- or high-oxygen conditions. This approach provided high-throughput protein identification and high-accuracy quantification. The approach also overcame some limitations of traditional gel-based proteomic approaches (such as the inability to detect highly acidic/basic/hydrophobic proteins or proteins of very high/low molecular weights), enabling us to achieve the best coverage of the *Drosophila* mitochondrial proteome to date, including proteins with a wide spectrum of pI and molecular weight values (Table S1) [42]. Furthermore, the use of the iTRAQ reagents improved the signal-to-noise ratio while increasing the signal intensity of the peptides, facilitating the detection and identification of low-abundance proteins [43], [44]. In addition, multiple iTRAQ-labeled proteins were analyzed in parallel, and the protein identification and quantification were based on multiple-peptide matching. This approach provided more consistent, accurate protein quantification results than do 2-DE-based analyses [45]. In the current study, we isolated mitochondria from the thoracic muscle and labeled the digested peptides with iTRAQ. Qualitative and quantitative protein signals were acquired by LC-MS/MS, which was followed by a statistical evaluation to identify the proteins that were differentially expressed in the three groups. A total of 718 proteins were identified in all of the *Drosophila* strains. To our knowledge, the current study provides the excellent coverage of the *Drosophila* mitochondrial proteome.

We have determined the overall characteristics of the proteomic alterations and detected a mild change in protein abundance in the LOF and HOF mitochondria (55 in LOF (7.7% of the identified proteins) and 75 in HOF (10.4% of the identified proteins)). The correlation between the proteins and their corresponding mRNA levels suggested that transcriptional, translational, and post-translational mechanisms are all involved in regulating protein abundance in response to extreme oxygen conditions. The abundance of typical mitochondrial proteins was regulated primarily at the transcriptional level. In contrast, the membrane-associated proteins were largely controlled by translational or post-translational mechanisms, such as protein translocation and/or stability. Indeed, previous studies have demonstrated that extreme oxygen conditions significantly impact both transcription and translation. For example, hypoxia-induced factors (HIFs) stimulate transcription under hypoxic conditions [46], and prolyl hydroxylases (PHDs) can regulate protein stability in response to altered oxygen tension [47].

The majority of the differentially expressed proteins in LOF that were associated with a subset of functions (such as calcium regulation, detoxification, glycolysis, chaperon, and antioxidant and translational regulation) exhibited reduced abundances compared with the control flies. However, a number of the differentially expressed proteins in LOF that were involved in respiration or associated with the mitochondrial membrane displayed increased abundances. The proteins that were differentially expressed in the HOF samples and were implicated in glycolysis, the TCA cycle, and respiration primarily displayed reduced abundances compared to the control flies, and most of the differentially expressed proteins in the remaining functional groups showed increased abundances. The changes in the respiratory chain complexes in the LOF or HOF could be explained in part by the observation of changes in protein abundance. Indeed, we identified and quantified approximately 62% of the components of the respiratory chain complexes, including 67% (32/48) of complex I, 43% (3/7) of complex II, 77% (10/13) of complex III, and 44% (8/18) of complex IV components. Of the detected complex I components, 97% (31/32) of the proteins exhibited no significant change in abundance in the LOF samples. In contrast, 66% (21/32) of the proteins showed dramatic decreases in abundance in the HOF samples. The protein abundance changes in the LOF or HOF samples were clearly consistent with the corresponding changes in respiration complex I activity (Figure 1H). Of the components detected in complex II, 2 of the 3 identified proteins were down-regulated in HOF; however, all of these proteins remained unchanged in LOF. The protein abundance changes therefore partially accounted for the decreased activity of complex II in HOF, but they failed to match the activity change in LOF, suggesting that the decreased complex II activity in the LOF samples may be regulated via post-translational protein modifications. Intriguingly, only a few of the components of respiratory chain complex III and IV detected in the current study showed changes in abundance in LOF or HOF; only 20% (2/10) of complex III and 25% (2/8) of complex IV components exhibited differences in protein expression. In most cases, the protein abundance changes were consistent with the activity of the corresponding complex. In LOF, the protein abundance changes in both complex III and complex IV were positively correlated with the corresponding respiratory chain activities. In HOF, the complex IV components displayed similar protein abundance and complex activity compared with the controls.

Furthermore, multiple studies have demonstrated that hyperoxia can stimulate the production of ROS in the mitochondria [3], [48], [49] at respiratory chain complexes I and III (predominantly complex I) [50], [51]. This increased mitochondrial ROS production is a key factor that introduces cell injury or death under hyperoxic conditions [52]–[54]. In HOF, the down-regulation of respiratory chain complexes I and II proteins may reduce the overall cellular respiration activity and decrease ROS generation. Indeed, previous studies have demonstrated that ROS generation was significantly lowered in mitochondria isolated from HOF samples [15], demonstrating that decreases in respiratory activities of complexes I, II and III play an important role in hyperoxia tolerance in this *Drosophila* strain. Further, current study revealed that HOF have evolved a mechanism to reduce ROS generation under hyperoxic conditions through the decreased expression of respiratory chain complexes I and II, demonstrating that quantitative proteomic data can establish a solid basis from which to elucidate the mechanisms underlying changes in the respiration capacities of LOF and HOF mitochondria. However, there was only a small overlap between the differentially expressed mitochondrial proteins in LOF and HOF, indicating that hypoxia- and hyperoxia-evoked responses in the mitochondrial proteome are largely distinct from each other.

We identified 15 proteins with changes in protein abundance in both LOF and HOF mitochondria. By searching an RNAi database containing more than 100 genome-wide functional RNAi screen results from *Drosophila* [55], we found that these commonly altered proteins were strongly enriched in screens of modulators of calcium (6 out of 15) [56] and Notch signaling (4 out of 15) [57] (Table 1), demonstrating that calcium signaling and Notch signaling pathways may play important roles in the adaptation to extreme oxygen conditions. Indeed, although the role of calcium signaling in the tolerance of hypoxia or hyperoxia remains unclear, our previous study demonstrated that Notch signaling is critical for adaptation to hypoxia [14], [58]. One of the most striking discoveries of the current study is the opposite changes in the expression of mitochondrial calcium transporters/channels in the LOF and HOF samples. Calcium is an important signaling molecule that is involved in the regulation of many cellular functions, such as ATP generation, redox control, and apoptosis [59], [60]. The large free energy in the calcium ion membrane gradients makes calcium signaling very sensitive to changes in cellular free energy, primarily ATP. Previous studies have demonstrated that mitochondria are the primary sources of aerobic energy production and maintain large calcium gradients across their inner membranes. Changes in calcium- transporting mechanisms in the LOF and HOF mitochondria suggest that cellular adaptation to extreme oxygen conditions requires the adjustment of cytosolic calcium homeostasis; this adjustment is likely achieved, at least in part, through the regulation of calcium storage and release in mitochondria. Furthermore, because the mitochondrial calcium level regulates NADH generation and oxidative phosphorylation, including the activities of the F_1_F_O_-ATPase and the cytochrome chain, such changes in LOF and HOF may also reflect an important mechanism of mitochondrial adaptation with respect to energy homeostasis and turnover under hypoxic or hyperoxic conditions. This change may optimize the efficiency of coupling and ATP production and reduce ROS generation in these flies [6], [13], [15]. Our results also suggest that crosstalk between the mitochondrial, calcium and Notch signaling pathways may be a key mechanism through which organismal development is regulated in an environment with an altered oxygen supply.

**Table 1:** List of differentially expressed proteins identified in both LOF and HOF samples.

			Fold Change			
Protein_ID	Annotation	Symbol	LOF	HOF	p value	Modulator of
FBpp0087838	CG8696	Mal-A1	0.73±0.10	0.68±0.05	<0.05	Notch signaling
FBpp0085448	CG15908	CG15908	0.53±0.05	0.65±0.03	<0.05	calcium signaling
FBpp0261451	CG33950	CG33950	1.30±0.12	1.35±0.09	<0.05	calcium signaling
FBpp0079400	CG3747	Eaat1	1.53±0.15	1.55±0.12	<0.05	ND
FBpp0082514	CG4264	Hsc70-4	1.35±0.06	1.43±0.08	<0.05	Notch signaling
FBpp0076522	CG7409	CG7409	1.68±0.19	3.38±0.44	<0.05	Notch signaling
FBpp0086062	CG14482	CG14482	1.43±0.05	1.48±0.03	<0.05	ND
FBpp0088986	CG5730	AnnIX	1.50±0.04	1.60±0.04	<0.05	ND
FBpp0077773	CG2718	Gs1	1.45±0.06	1.40±0.06	<0.05	ND
FBpp0076103	CG14168	CG14168	1.88±0.25	1.55±0.14	<0.05	calcium signaling
FBpp0111540	CG34387	futsch	2.18±0.22	1.45±0.06	<0.05	calcium signaling
FBpp0072124	CG3725	Ca-P60A	0.65±0.05	1.53±0.09	<0.05	calcium signaling
FBpp0080637	CG10302	bsf	0.58±0.03	1.53±0.06	<0.05	ND
FBpp0082295	CG9297	CG9297	0.63±0.03	1.45±0.06	<0.05	calcium/Notch signaling
FBpp0085482	CG3152	Trap1	0.65±0.03	1.75±0.09	<0.05	ND

**Note:**

Portein_IDs were from FlyBse (flybase.org).

LOF: Hypoxia tolerant fly sample.

HOF: Hyperoxia tolerant fly sample.

Fold Changes were shown as (mean ± SEM).

p values were from Mann Whitney Test.

ND: not Detected.

In summary, we analyzed the proteomes of mitochondria isolated from *Drosophila melanogaster* that were adapted to extremely low- or high-oxygen conditions. We believe that our findings will not only provide a better understanding of the mechanisms underlying adaptation to extreme oxygen conditions in *Drosophila* but will also hope to provide a clue in studying human disease induced by altered oxygen tension in tissues and cells.

## Supporting Information

Figure S1 **The distribution of proteins with different spectra abundance group across protein lengths groups.** Proteins were divided to 5 groups by their length (length≦100, 100<length≦500, 500<length≦1000, 1000<length≦5000, 5000<length≦10000) in every spectra group (spectra>50, 10≦spectra≦50, spectra<10). The percentages of the proteins in each length group against the sum of these proteins were achieved (number of proteins in each length group divided by total numbers of the proteins in corresponding spectra group).
(TIF)

Table S1 List of proteins identified and quantified by the iTRAQ analysis.
(XLSX)

Table S2 List of peptides identified in the first injection.
(XLSX)

Table S3 List of peptides identified in the second injection.
(XLSX)

Table S4 List of peptides identified in both injections.
(XLSX)

Table S5 Summary of differentially expressed proteins identified in LOF.
(XLSX)

Table S6 Summary of differentially expressed proteins identified in HOF.
(XLSX)

Table S7 Summary of differentially expressed proteins both in LOF or HOF.
(XLSX)

Table S8 Scatter plot correlation analysis of the differentially expressed proteins and corresponding mRNAs in LOF and HOF.
(XLSX)

Table S9 Scatter plot correlation analysis of the differentially expressed proteins and corresponding mRNAs in certain functional groups.
(XLSX)

Table S10 List of proteins in TCA cycle, Respiratory Chain Complexes and ATP synthase identified by iTRAQ analysis.
(XLSX)
