# Supplementary figures and images for: Quantitative Evaluation of the Mitochondrial Proteomes of Drosophila melanogaster Adapted to Extreme Oxygen Conditions
Source: PLoS One. 2013 Sep 12;8(9):e74011. doi: 10.1371/journal.pone.0074011 (PMC3771901; doi:10.1371/journal.pone.0074011)

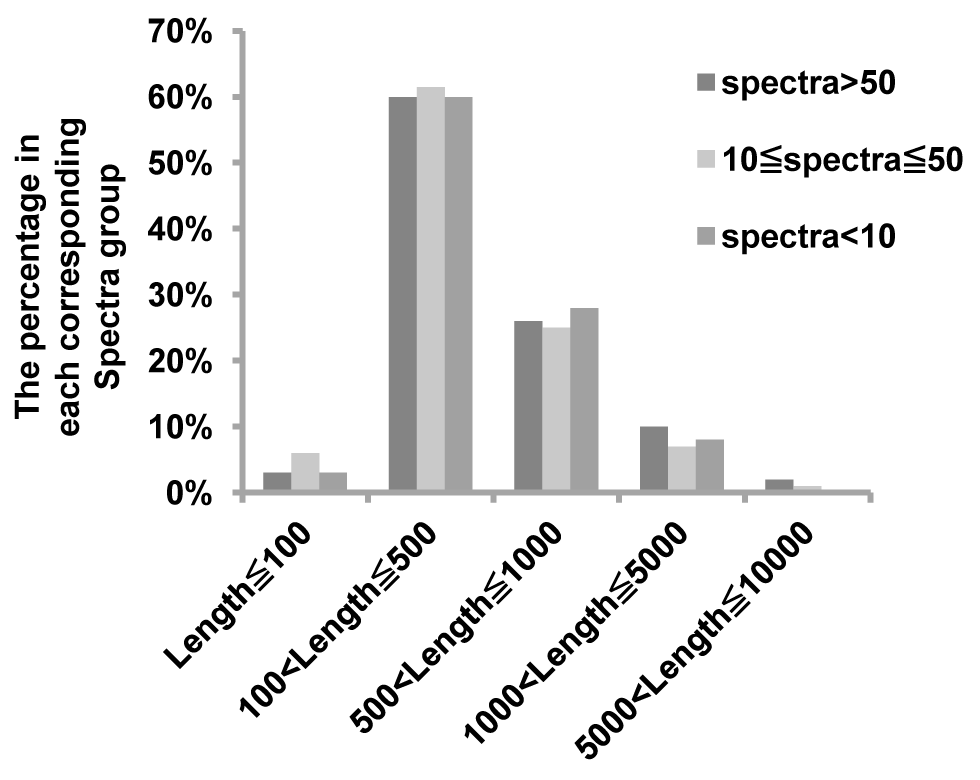

Supplement: Figure S1 — The distribution of proteins with different spectra abundance group across protein lengths groups. Proteins were divided to 5 groups by their length (length≦100, 100<length≦500, 500<length≦1000, 1000<length≦5000, 5000<length≦10000) in every spectra group (spectra>50, 10≦spectra≦50, spectra<10). The percentages of the proteins in each length group against the sum of these proteins were achieved (number of proteins in each length group divided by total numbers of the proteins in corresponding spectra group). (TIF) [file pone.0074011.s001.tif]
